# A Phase Two Randomised Controlled Double Blind Trial of High Dose Intravenous Methylprednisolone and Oral Prednisolone versus Intravenous Normal Saline and Oral Prednisolone in Individuals with Leprosy Type 1 Reactions and/or Nerve Function Impairment

**DOI:** 10.1371/journal.pntd.0001041

**Published:** 2011-04-12

**Authors:** Stephen L. Walker, Peter G. Nicholls, Sushmita Dhakal, Rachel A. Hawksworth, Murdo Macdonald, Kishori Mahat, Shudan Ruchal, Sushma Hamal, Deanna A. Hagge, Kapil D. Neupane, Diana N. J. Lockwood

**Affiliations:** 1 Department of Clinical Research, Faculty of Infectious and Tropical Diseases, London School of Hygiene and Tropical Medicine, London, United Kingdom; 2 School of Health Sciences, University of Southampton, Southampton, United Kingdom; 3 Anandaban Hospital, Kathmandu, Nepal; Kwame Nkrumah University of Science and Technology (KNUST) School of Medical Sciences, Ghana

## Abstract

**Background:**

Leprosy Type 1 reactions are a major cause of nerve damage and the preventable disability that results. Type 1 reactions are treated with oral corticosteroids and there are few data to support the optimal dose and duration of treatment. Type 1 reactions have a Th1 immune profile: cells in cutaneous and neural lesions expressing interferon-γ and interleukin-12. Methylprednisolone has been used in other Th1 mediated diseases such as rheumatoid arthritis in an attempt to switch off the immune response and so we investigated the efficacy of three days of high dose (1 g) intravenous methylprednisolone at the start of prednisolone therapy in leprosy Type 1 reactions and nerve function impairment.

**Results:**

Forty-two individuals were randomised to receive methylprednisolone followed by oral prednisolone (n = 20) or oral prednisolone alone (n = 22). There were no significant differences in the rate of adverse events or clinical improvement at the completion of the study. However individuals treated with methylprednisolone were less likely than those treated with prednisolone alone to experience deterioration in sensory function between day 29 and day 113 of the study. The study also demonstrated that 50% of individuals with Type 1 reactions and/or nerve function impairment required additional prednisolone despite treatment with 16 weeks of corticosteroids.

**Conclusions:**

The study lends further support to the use of more prolonged courses of corticosteroid to treat Type 1 reactions and the investigation of risk factors for the recurrence of Type 1 reaction and nerve function impairment during and after a corticosteroid treatment.

**Trial Registration:**

Controlled-Trials.comISRCTN31894035

## Author Summary

Leprosy is caused by a bacterium and is curable with a combination of antibiotics known as multi-drug therapy that patients take for six or 12 months. However a significant proportion of leprosy patients experience inflammation in their skin and/or nerves, which may occur even after successful completion of multi-drug therapy. These episodes of inflammation are called leprosy Type 1 reactions. Type 1 reactions are an important complication of leprosy because they may result in nerve damage that leads to disability and deformity. Type 1 reactions require treatment with immunosuppressive agents such as corticosteroids. The optimum dose and duration of corticosteroid therapy remains unclear. We conducted a study to see if it would be safe to use a large dose of a corticosteroid called methylprednisolone for three days at the start of a 16 week corticosteroid treatment regime of prednisolone in patients with leprosy Type 1 reactions and leprosy patients with nerve damage present for less than six months. We did this by comparing individuals who were given methylprednisolone followed by prednisolone and those who received just prednisolone. In this small study we did not see any significant difference in the frequency of adverse events due to corticosteroid treatment in the two groups. We did not demonstrate a significant difference in improvement in individuals in the methylprednisolone group (who received a larger dose of corticosteroids) than those in the prednisolone treated group. Overall approximately 50% of individuals required more prednisolone in addition to the 16 week course of treatment to prevent further nerve damage or reactions. This suggests that it would be worthwhile investigating longer treatment courses with corticosteroids and other immunosuppressive drugs.

## Introduction

Leprosy is a chronic granulomatous infection principally affecting the skin and peripheral nerves caused by the obligate intracellular organism *Mycobacterium leprae* [2]. The disease causes skin lesions and neuropathy. Complications secondary to the neuropathy can result in deformity and disability. 249 007 new cases of leprosy were diagnosed and reported to World Health Organization (WHO) in 2008 [3].

Type 1 reactions (T1Rs) are a major cause of nerve function impairment (NFI) in patients with leprosy and affect up to 30% of susceptible individuals [4]. T1Rs predominantly affect borderline leprosy[4]. They may be a presenting feature of leprosy or occur during multi-drug therapy (MDT) or after completion. A T1R is characterised by acute inflammation in skin lesions or nerves or both. Skin lesions become acutely inflamed and oedematous and may ulcerate. Oedema of the hands, feet and face can also be a feature of a T1R.

Leprosy T1Rs are treated with oral corticosteroids. However treatment with a standardised reducing 12 week course of oral prednisolone (total dose 1.68 g) which had been used in a previous pilot study in Nepal resulted in 37% of individuals requiring additional prednisolone [5]. The randomised controlled treatment trials TRIPOD 2 and TRIPOD 3 that were reported during the design of this study had both used a 16 week course of oral prednisolone (total dose 2.52 g) [6], [7].

T1Rs appear to be mediated via Th1 type cells and lesions in reaction express the pro-inflammatory IFNγ, IL12 and the oxygen free radical producer iNOS [8]. The expression of TNFα protein in the skin and nerves of patients during T1Rs is increased[9]. High dose intravenous (IV) methylprednisolone (MP) has been used as a standard treatment in the early phase of an exacerbation of Th1 cytokine mediated relapsing chronic diseases. These conditions include rheumatoid arthritis (RA) [10] and multiple sclerosis (MS) [11]. In 18 patients with MS treated with IV MP 1 g for three days there was a significant suppression of mitogen stimulated IFNγ, TNFα and IL2 production by blood leucocytes ex vivo after treatment [12]. MP has also been shown to reduce serum levels of TNFα in RA [13]. Eleven patients given 1 g IV showed significantly reduced serum levels of TNFα at 4 and 24 hours. In a comparative study of lymphocyte-suppressive potency between prednisolone and MP in 44 individuals with RA the latter was more effective in those with greater disease activity as defined by rheumatoid factor titres [14].

We compared three daily infusions of IV high dose MP and oral prednisolone with a 16 week course of oral prednisolone alone. High dose IV MP had not been used previously in a trial of treatment of leprosy T1R so a Phase 2 trial was needed to confirm safety before considering whether to proceed to a larger Phase 3 trial of clinical efficacy.

The aims of the trial were as follows:

1. To assess the safety and tolerability of high dose MP when given to patients with leprosy T1Rs and patients with leprosy associated acute neuritis with nerve function impairment in Nepal.
2. To assess the effect of high dose MP on the clinical outcome of leprosy T1Rs and leprosy associated acute neuritis with nerve function impairment.

## Methods

A double blind parallel-group randomised controlled trial was designed to compare the safety and effect of early high dose IV MP followed by oral prednisolone with IV Normal saline and oral prednisolone. The study was approved by the Nepal Health Research Council and the Ethics Committee of the London School of Hygiene and Tropical Medicine (Number 4022).

Participants (aged between 16–65 years and weighing more than 30 kg) were recruited from the leprosy service of Anandaban Hospital, Kathmandu, Nepal. Two groups of individuals were eligible for entry into the trial:

1. Individuals diagnosed as having leprosy with clinical evidence of T1R of less than six months duration.
2. Individuals diagnosed with leprosy with new (less than six months duration) NFI without inflammation of skin lesions (if skin lesions were present).

Participants with any type of leprosy of the Ridley-Jopling Classification [15] were eligible. Initially, enrolment into the study required individuals with clinical evidence of a T1R to have associated nerve function impairment. This was changed nine months after the start of the trial so that individuals with T1Rs involving the skin only would also be eligible for enrolment. This was done because only 14 individuals had been recruited in this period and recruitment had been optimal as determined by case note review of a random selection of clinic attendees. The change to this eligibility criterion was approved by the two Ethics committees.

The following individuals were excluded: those unwilling to give consent or return for follow-up or who had taken systemic steroids within three months of enrolment, those who had received other immunosuppressant therapy including thalidomide within three months of enrolment, those with severe active infection such as tuberculosis or severe intercurrent disease, those with a contraindication to high dose methylprednisolone such as peptic ulcer disease, diabetes mellitus, glaucoma and uncontrolled hypertension or known allergy to methylprednisolone. Pregnant women were excluded and females of child bearing capacity were not recruited unless they had at least one month of adequate contraception.

The participants were treated with corticosteroids for 112 days. The total duration of the study was 337 days from entry into the trial. The intervention for the MP treated individuals was 1 gram MP in Normal saline given as an IV infusion and eight dummy tablets (Comprehensive Medical Services India, Chennai India) identical in appearance to prednisolone tablets daily for the first three days of the trial. The prednisolone treated individuals received 40 mg (eight tablets) of prednisolone and an identical appearing IV infusion which contained only Normal saline daily for the first three days of the trial. Thereafter individuals in both groups received the same reducing course of prednisolone. This course was prednisolone 40 mg daily from day 4 to day 14 of the study. The amount of prednisolone was then reduced to 35 mg daily for the next 14 days and then by a further 5 mg daily every 14 days to zero. An individual allocated to the MP group received a total dose of corticosteroid equivalent to 6.15 g of prednisolone. Individuals in the prednisolone alone group received 2.52 g of prednisolone in total.

All individuals enrolled into the study received albendazole 400 mg daily for the first three days of the trial and famotidine 40 mg daily whilst they were receiving corticosteroids. The albendazole was given to reduce the risk of hyperinfection with *Strongyloides stercoralis*. The famotidine was used to reduce the risk of peptic ulceration.

The primary outcome measure was the frequency of adverse events in the two treatment arms. These were assessed by a study physician prior to treatment and then at day 4 (after the three IV infusions) and then days 8, 15, 29, 57, 85, 113, 141, 169, 197, 225, 253, 281, 309 and 337. Adverse events were enquired about and examined for at each assessment. A standardized form contained a list of adverse events attributable to corticosteroids which participants were asked if they had experienced. There was also a free text space available where other symptoms mentioned by the study participants or identified by the physician could be recorded. Adverse events were defined as major or minor in accordance with the classification used in the TRIPOD studies [16]. Major adverse events were defined as psychosis, peptic ulcer, glaucoma, cataract, diabetes mellitus, severe infections (including tuberculosis), infected neuropathic ulcers, hypertension and death. Minor adverse events were defined as moon face, dermatophyte fungal or yeast infections, acne and gastric pain requiring an antacid (in addition to the famotidine each individual was prescribed whilst on corticosteroids). Individuals were questioned about the symptoms of nocturia, polyuria and polydipsia as a method of screening for diabetes mellitus in addition to urinalysis being performed.

Secondary outcomes measures were:

- change in the clinical severity score derived from the validated Clinical Severity Scale [17] at days 4, 29, 113 and 337. The Clinical Severity Scale uses a composite score of skin signs and oedema, sensory and motor nerve function[17]. We had previously developed the scale and demonstrated that it has a Cronbach's alpha of >0.8 and an Intra-Class Correlation coefficient of 0.994.
- change in clinical nerve function impairment determined using the validated Clinical Severity Scale at days 4, 29, 113 and 337.
- time to the next steroid requiring reactional episode or acute nerve function impairment.
- the amount of supplementary prednisolone required in addition to the reducing 16 week regimen.

Peripheral nerve function was assessed clinically. Sensory testing (ST) was performed using two Semmes-Weinstein monofilaments (SWM) (Sorri-Bauru, Bauru, São Paulo, Brazil) at designated test sites on the hands and feet as previously reported [17]. The sensation in the areas of skin supplied by the ulnar and median nerves was tested with 2 g and 10 g monofilaments. The area of skin supplied by the posterior tibial nerve was tested with the 10 g and 300 g monofilaments. Trigeminal nerve sensation was tested using cotton wool. Voluntary muscle testing (VMT) was assessed using the modified Medical Research Council grading of power [18]. The facial nerve was tested by assessing forced eye closure. The median nerve was tested using resisted thumb abduction, the ulnar nerve by resisted little finger abduction and the radial nerve by resisted wrist extension. The lateral popliteal nerve was tested by resisted foot dorsiflexion. ST and VMT assessments were carried out by trained physio-technicians and if necessary repeated by the study physicians.

Patients with deterioration in nerve function or skin signs were treated with further prednisolone. This was defined as a sustained deterioration (for a period of at least two weeks) of nerve function, the development of nerve pain unresponsive to analgesics, palpable swelling of skin patches or new erythematous and raised skin patches. Any decline in nerve function which the study doctors believed required immediate additional prednisolone was also regarded as deterioration. Individuals who experienced deterioration in skin and/or nerve function whilst receiving a dose of prednisolone less than 20 mg daily had the dose increased back to 20 mg and reduced by 5 mg every 14 days to zero. The exception to this was if they had a T1R involving a facial patch in which case the prednisolone was increased to 40 mg regardless of the dose of prednisolone the individual was taking. Individuals taking a dose of prednisolone greater than 20 mg had their dose increased to 40 mg and tapered by 5 mg every 14 days to zero.

In order to have 80% power to show that MP was not associated with a significantly greater (α<0.05) rate of major adverse effects it was calculated that the study would need 201 participants in each group based on a higher rate of 7%. Using this same assumption but with the TRIPOD data for all the Nepali participants (major adverse effect rate of 2.4%) then 64 individuals would be needed to be enrolled in each arm.

Eligible individuals were enrolled consecutively. Block randomisation in groups of four using a table of random numbers generated by Dr Peter Nicholls was used. A standard envelope system was used for allocation concealment. The envelopes were pre-packed in London by Dr Claire Watson who had no other involvement with the study. The participants were randomly allocated to the MP/prednisolone or the prednisolone alone arm and so had an equal chance of being in either arm of the study. The allocation procedure was decentralized and operated solely by the chief pharmacist at Anandaban Hospital who kept a separate record of the allocation. The pharmacist had no contact with the study participants during their inpatient stay.

All study participants, physicians, ward staff and other assessors (physio-technicians) were blinded to the allocation. Only Dr Peter Nicholls had access to the study data and the randomisation code. The allocation code was revealed to the other researchers once recruitment, follow-up and data collection had been completed.

The data were stored in an Access database and analysed using the Statistical Package for the Social Sciences (SPSS version 16 SPSS Inc., Chicago, Illinois). An intention to treat analysis was used for calculating the effects of treatment on individuals in each group.

The trial was registered with Current Controlled Trials Ltd (www.controlled-trials.com) in accordance with the policy of the International Committee of Medical Journal Editors [1] and was assigned the unique identifier ISRCTN31894035. The protocol for the trial can be accessed as a supplementary file Protocol S1 to this publication.

## Results

**Figure 1:**
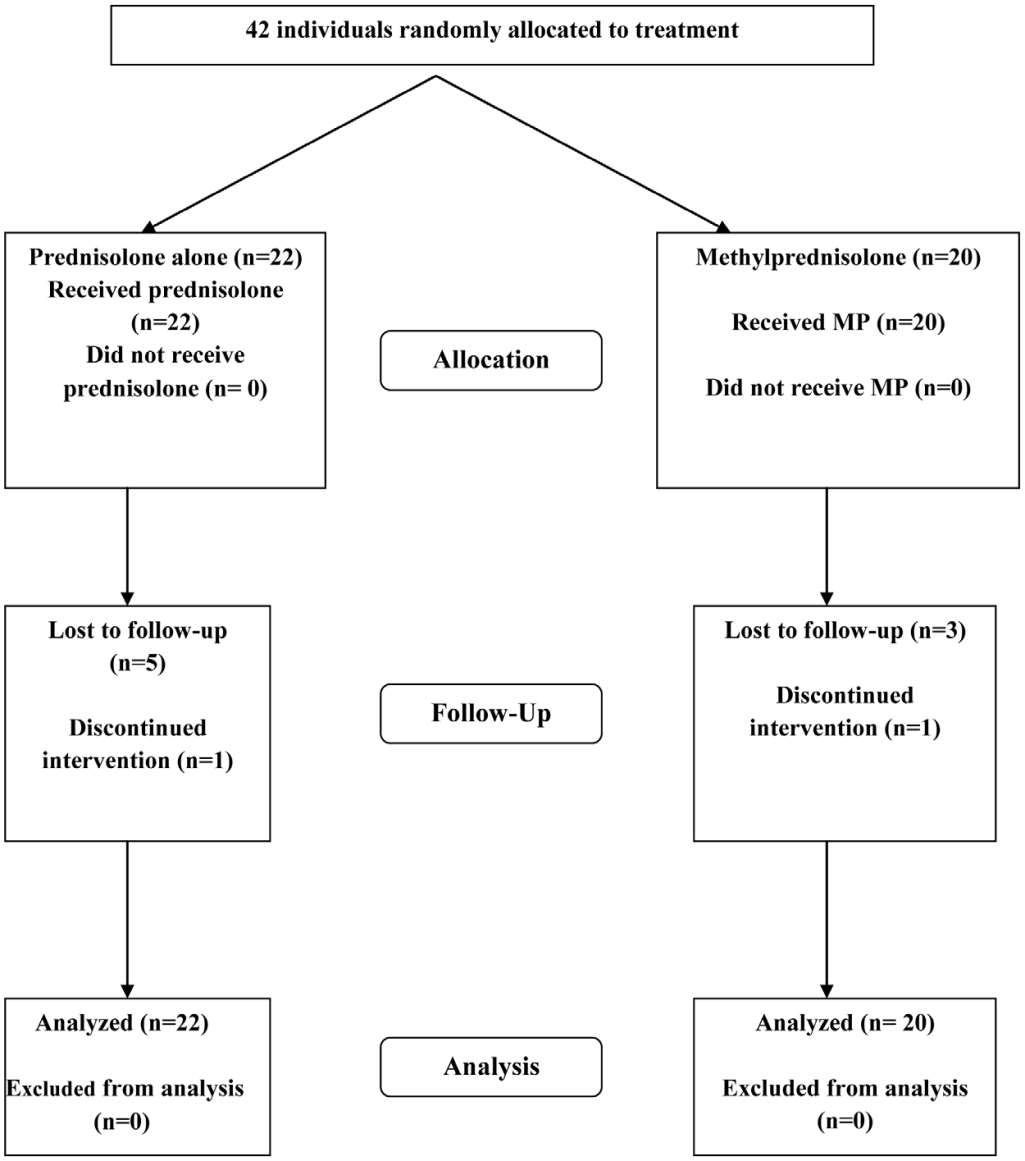
CONSORT flow diagram for the pilot study of individuals randomized to either intravenous methylprednisolone and oral prednisolone or oral prednisolone alone.

Forty-two patients were enrolled into the trial between 7^th^ December 2005 and 31^st^ December 2007. The final assessment and data entry was completed on 5^th^ November 2008. The participants flow through the study is illustrated in the CONSORT flow diagram (Figure 1).

Thirty-three males and nine females were recruited. Twenty-two individuals were randomised to receive prednisolone only. There were no statistically significant differences between the groups with respect to gender, age, Ridley-Jopling classification, or treatment with MDT (Table 1). The two groups did not differ significantly in terms of the nature of the reaction, the type of NFI at baseline or the pattern of old (> 6 months duration) NFI.

**Table 1:** Baseline characteristics of study participants in each arm.

		PREDNISOLONE (n = 22)	METHYLPREDNISOLONE (n = 20)
Sex	Female	5	4
	Male	17	16
Median Age [years (Range; min-max)]	Female	39 (19;35–54)	17.5 (25;17–42)
	Male	40 (43;22–65)	28.5 (48;16–64)
Ridley-Jopling classification	Tuberculoid	0	1
	Borderline tuberculoid	11	12
	Borderline borderline	0	3
	Borderline lepromatous	10	3
	Lepromatous leprosy	1	1
Reaction Type	Skin Only	4	4
	Skin and Nerves	8	13
	Nerves Only	10	3
MDT Status	Untreated	3	5
	On treatment	14	10
	Treated	5	5

Eight participants (19%) did not complete the full schedule of follow-up. Five were randomised to the prednisolone arm and three received MP. Efforts were made to get these individuals to attend by telephoning or writing to them but without success. Two of these individuals stopped attending whilst on corticosteroids.

Table 2 shows the number of individuals who experienced a particular adverse event. Twenty-three participants experienced at least one adverse event, twelve (54.5%) in the prednisolone arm and 11 (55%) in the MP arm. Seven individuals experienced more than one adverse event. There were no statistically significant differences in the number of individuals experiencing a given adverse event between the two groups of the study.

**Table 2:** Minor and major adverse events.

Adverse Event		Prednisolone	Methylprednisolone	chi square (Fisher's exact)
Minor				
	Moon Face	2	6	0.123
	Acne	5	5	1
	Fungal infection	0	1	0.476
	Gastric pain	5	2	0.414
	NPP	2	2	1
	Weight gain	1	0	1
Major	Glaucoma	1	0	1
	Infected ulcers	0	1	0.476

Two individuals (one from each arm of the study) experienced a major adverse event. One was diagnosed with glaucoma and the other with infected neuropathic ulcers. None of the participants developed hypertension, tuberculosis or diabetes mellitus. The risk ratio of having an adverse event (of any type; major or minor) given that the participant received MP was 1.0083 (95% CI: 0.5817 to 1.7480; p = 0.9764) compared to prednisolone.

**Figure 2:**
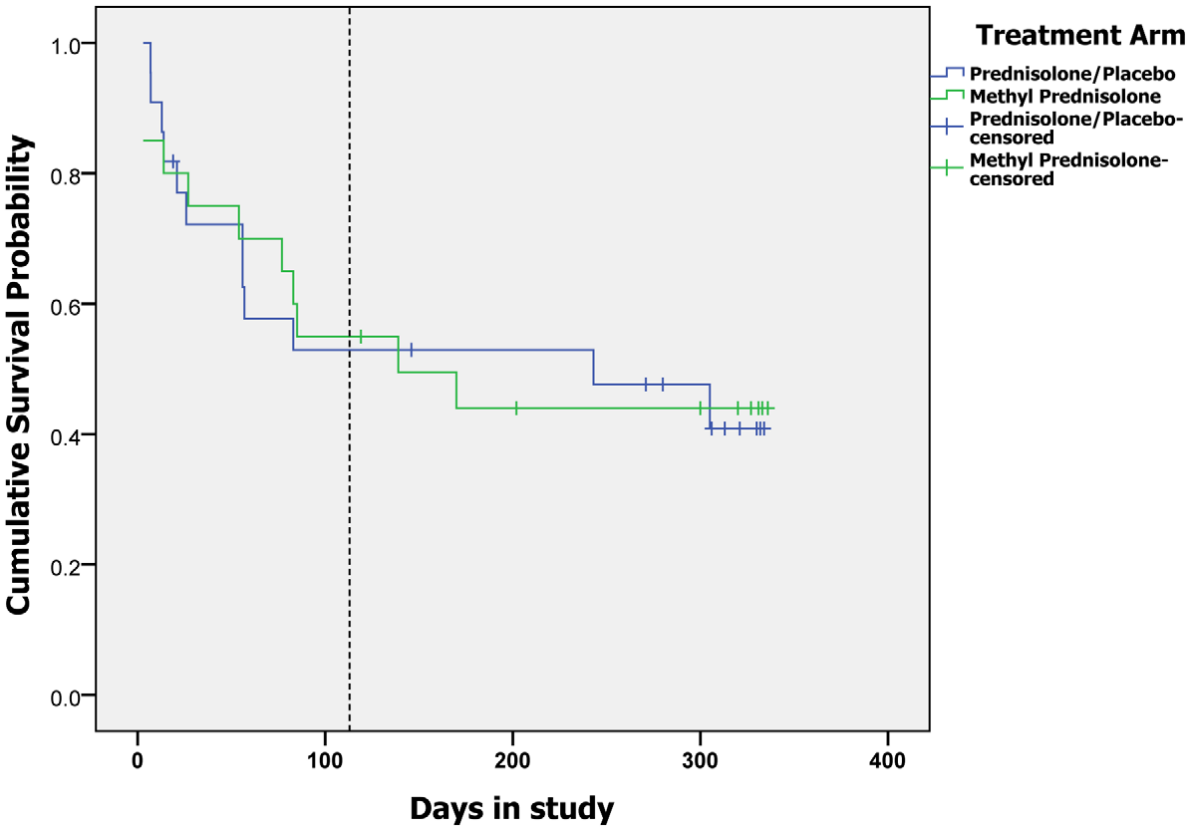
Time to first adverse event. (The vertical broken line is placed at day 113).

Individuals were most likely to experience an adverse event whilst taking the first course of corticosteroids between days 1 and 112. Figure 2 is a Kaplan-Meier survival curve showing the cumulative “survival” probability (i.e. not having an adverse event) for individuals in each group. There was no significant difference between the two groups (Log Rank (Mantel-Cox) 0.945).

Four individuals had their first adverse event after the initial study intervention had been completed (post day 112). Two others had a new adverse event after the intervention period. Two individuals experienced an adverse event, weight gain and infected neuropathic ulcers respectively, whilst not taking corticosteroids.

**Figure 3:**
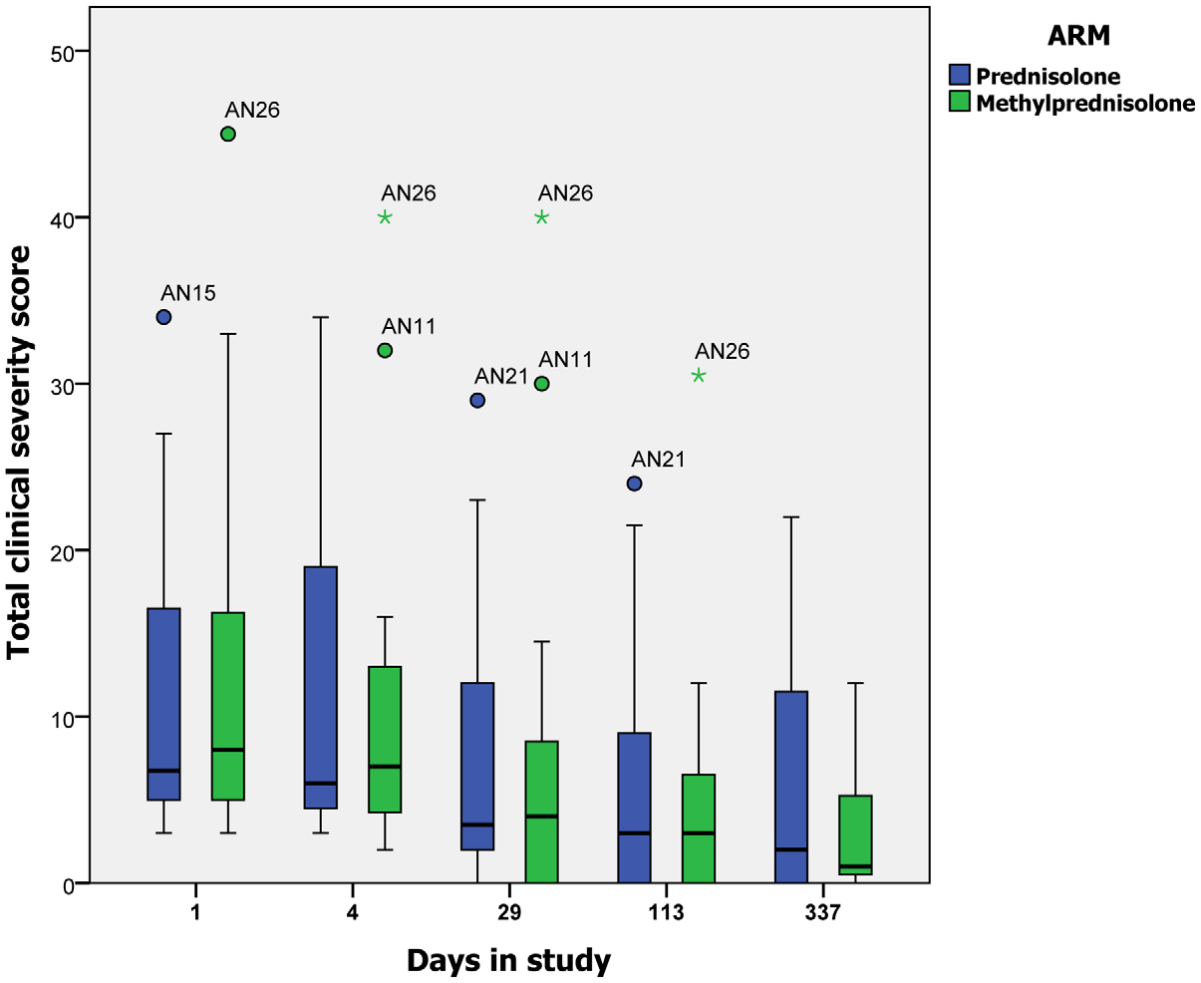
Total severity score at days 1, 4, 29, 113, 337 (Circles denote individuals 1.5 times the interquartile range (IQR) outside the box and asterisks denote individuals 3 times the IQR outside the box).

The total clinical severity scores, calculated using the validated scale, for each arm of the study at day 1 (enrolment) and days 4, 29,113 and 337 are shown using boxplots (fig.3). There was a downward trend in the total clinical severity scores of both groups. There were no statistically significant differences between the prednisolone and MP groups at any time point.

**Figure 4:**
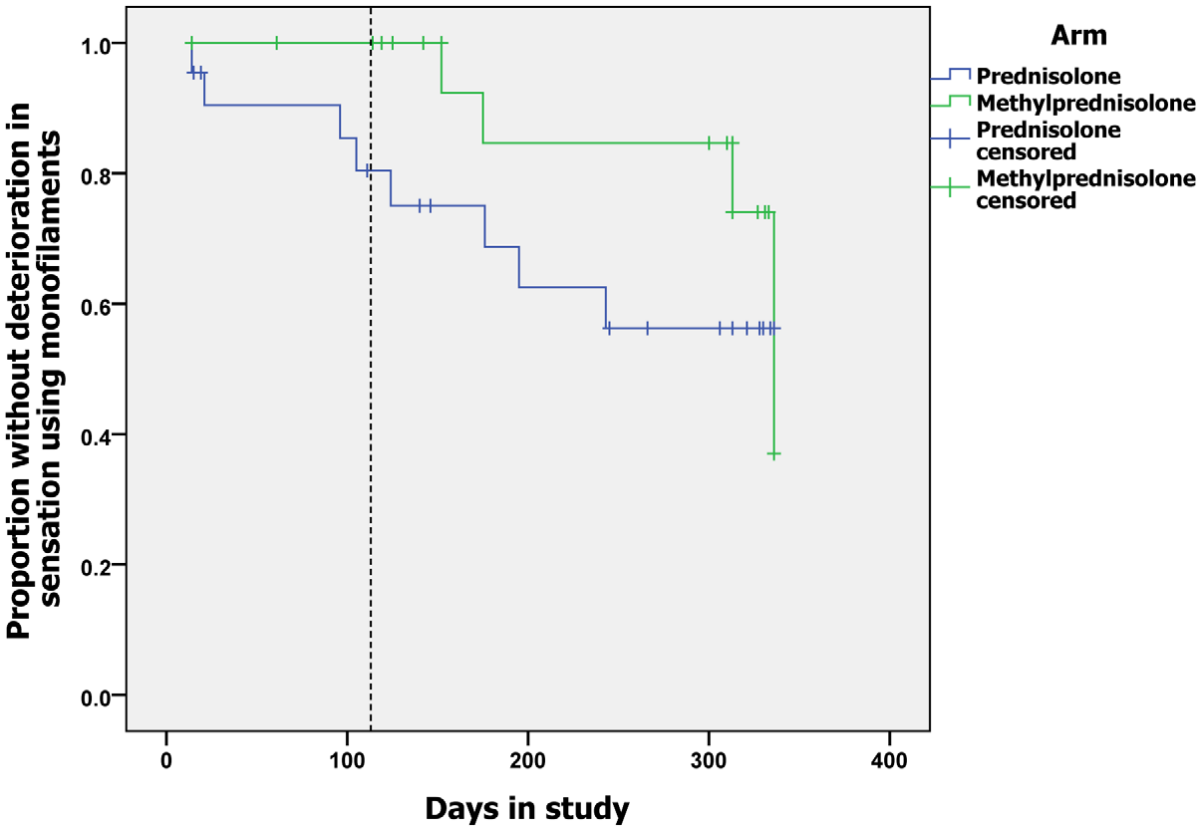
Time to deterioration of sensory function.

There was no significant difference in the median sensory scores (corrected for impairment >6 months) of individuals in the two groups at baseline. Both groups showed a downward trend in the sensory scores during treatment but there were no significant differences at any of the pre-specified time points. The Kaplan-Meier survival analyses of deterioration in sensory score during the study to days 29, 113 and 337 (fig.4) demonstrate that there is no difference between the groups at day 29 but at day 113 there was a significant difference in the probability of deterioration in sensation between individuals in the two arms of the study (p = 0.046). Patients in the prednisolone alone group were more likely to experience deterioration in sensation between day 30 and day 113. This effect is not maintained at the end of the study follow-up period at day 337. The motor scores of the two groups at baseline are not significantly different. They showed a downward trend during the course of the study. There are no significant differences between the scores of the group at any of the time points. There were no significant differences between the groups in the probability of an individual experiencing deterioration in motor function at days 29, 113 or 337.

**Figure 5:**
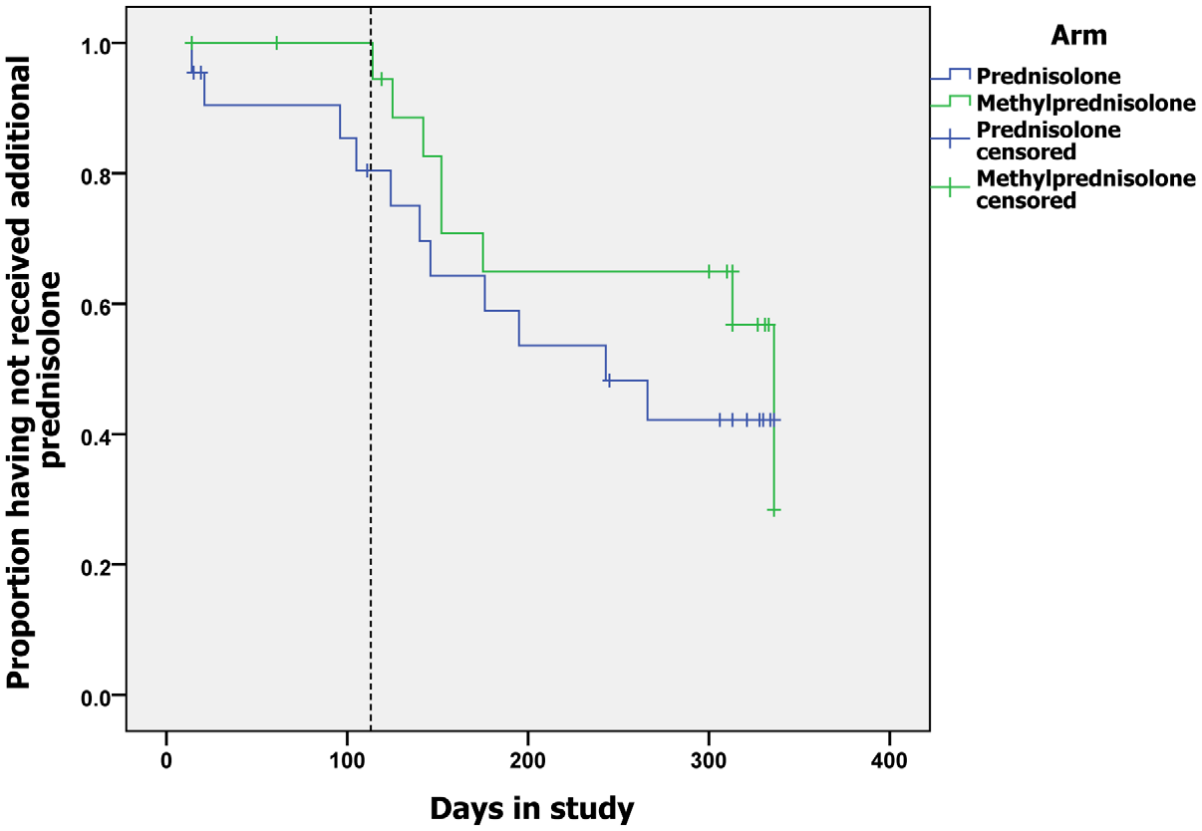
Time to requiring first course of additional prednisolone.

Figure 5 shows events when additional steroid was prescribed and censoring individuals who were unavailable for further assessment or who received prednisolone either inappropriately or for ENL. There was no significant difference in the probability of being prescribed additional prednisolone between the two groups (Log Rank (Mantel Cox) p = 0.126). The amount of additional prednisolone required by individuals randomised to either treatment group did not differ significantly. The mean amount of additional prednisolone prescribed during the study was 1252.5 mg (SD±1862.0) for the MP group and 1432.7 mg (SD±1245.9) for the prednisolone alone group (p = 0.718).

Twenty individuals (47.6%) required additional prednisolone because they experienced a deterioration of nerve function (n = 11) or a recurrence of a T1R (n = 6) or both (n = 3). Two individuals received additional prednisolone inappropriately and two developed ENL requiring prednisolone. Five of the 20 individuals (appropriately prescribed additional prednisolone for a trial indication) required prednisolone before day 112, the last day of the intervention period. The median time to requiring additional prednisolone for these individuals was 61 days (range = 14–105) after enrolment when individuals were receiving prednisolone 20 mg daily. The other 75% had finished the prednisolone before experiencing a deterioration requiring further treatment. The median number of days between finishing the study intervention (day 112) and requiring additional prednisolone was 63 days (range = 2–224).

Analysing the additional corticosteroid requirement by Ridley-Jopling classification fifty-two percent (12 of 23) of individuals with borderline tuberculoid (BT) leprosy, 67% (two of three) of individuals with borderline borderline (BB) leprosy, 38% (five of 13) of those with borderline lepromatous leprosy (LL) and 50% (one of two) of lepromatous leprosy patients required additional prednisolone for a trial indication (those with ENL were excluded). Two of the BT patients had positive slit-skin smears. The median time from enrolment to the deterioration requiring additional prednisolone was 152 days for BT patients, 138 days for BB patients, 125 days for BL patients and 313 days for those with LL. There were no significant differences in the proportion of individuals with a particular Ridley Jopling classification or the time to requiring additional prednisolone.

## Discussion

In this small, study the occurrence and timing of minor and major adverse events did not differ significantly between the prednisolone and the MP treated groups. The study was underpowered and limited the ability to detect significant differences of less than 30% between the groups. Twenty-one (50%) individuals experienced at least one minor adverse event and two (4.8%) a major adverse outcome. In the TRIPOD trials 8.4% (14/167) of the prednisolone treated Nepali cohorts experienced a minor adverse event[16]. This was not significantly different from the placebo treated group. The individuals in these groups were treated with either 1.96 g or 2.52 g of prednisolone depending on which of the three trials they were enrolled into.

The two major adverse events that occurred during the study were glaucoma and infected neuropathic ulcers but these were probably not due to the trial medications. One individual developed glaucoma at day 305. He developed ENL at day 111. ENL like corticosteroid therapy is a recognised cause of secondary glaucoma. He required continuous oral prednisolone (receiving a total additional dose of 2.87 g of prednisolone between days 111 and 305) despite treatment of his ENL with high dose (300 mg daily) clofazimine. The majority of individuals who develop ENL require long term treatment and many become corticosteroid dependent [19]. There were no cases of glaucoma in any of the TRIPOD participants. Infected neuropathic ulcers affected one individual treated with MP. This occurred 58 days after this man completed the trial intervention. Two individuals in the TRIPOD studies (one from the prednisolone treated group) developed infected ulcers. It is not reported whether the prednisolone treated person was taking the drug at the time the infection was diagnosed.

The symptoms of nocturia, polyuria and polydipsia were reported by four (9.5%) of individuals. The two individuals who had glycosuria did not complain of these symptoms. Their glycosuria was not persistent and therefore not considered to be clinically significant. The two individuals were both receiving additional prednisolone at the time but neither had received MP. There were no individuals in the study diagnosed with diabetes mellitus. The TRIPOD 1 study reported one individual from the prednisolone treated group who developed glycosuria. This was considered a major adverse event in that study but the authors did not comment whether this patient was diagnosed with diabetes mellitus [20]. Three individuals in the steroid treated groups of the three TRIPOD studies developed diabetes mellitus compared with one in the placebo groups but this difference was not significant [16].

The size of the study limited our ability to detect rare adverse events however a much higher rate of acne and moon face was recorded than the TRIPOD studies. Another factor that might have reduced our estimation of adverse events is the duration of follow-up which may have been too short, however most studies have assumed that adverse events will occur during the treatment phase predominantly. We were also unable to examine the effect of our interventions on bone density which may be significantly affected by corticosteroid therapy in the doses and durations commonly used to manage leprosy T1R and NFI. The findings would support the view that MP, in the doses used in the study, is safe.

MP did not appear to have a larger therapeutic effect than prednisolone alone on the symptoms and signs of leprosy T1Rs and NFI in this study. The use of a validated scale to measure leprosy T1Rs and NFI allows the comparison of the two groups in this study. There were no significant differences in the total severity score or the sensory or motor scores between the prednisolone and MP treated groups at any of the pre-defined time points. However there was a trend towards improvement in sensory and motor scores during the study. Participants in the prednisolone treated group were significantly more likely to have experienced deterioration in sensory function than the MP treated group by the end of the intervention (day 113). However this difference was not sustained to the end of the study. This effect may have occurred by chance as it was not reproduced in the skin or in motor function. The number of participants contributing to all of the survival analyses towards the end of the study is small and the results therefore less reliable. This phenomenon of deterioration after stopping corticosteroids is similar to the results of the TRIPOD 1 study of prednisolone given to patients as prophylaxis to prevent the occurrence of reactions and NFI. It demonstrated a protective effect of prednisolone compared with placebo during the 16 weeks of treatment which was lost by 48 weeks. The higher dose may have a greater effect whilst an individual is receiving corticosteroids but not once they are no longer taking the drug.

Forty-five per cent of the MP group and 50% of the prednisolone alone group were prescribed additional prednisolone. Of the 20 individuals who required additional prednisolone 12 (60%) did not do so until at least 28 days after completing the trial intervention. The clinical nature of the deterioration (skin or nerves or both) did not differ significantly between those who experienced it whilst receiving the study intervention and those who experienced deterioration after completing it (χ^2^ = 0.292). The delay in deterioration in the majority of individuals requiring additional prednisolone is similar to that seen in the TRIPOD 1 study[20].

After the start of this trial data suggesting that more prolonged courses of prednisolone may be more effective in treating T1Rs were published. The requirement for extra prednisolone was used as the outcome measure in the multi-centre double blind randomised controlled trial of three different prednisolone regimens conducted in India [21]. The proportion of individuals requiring additional prednisolone in the three groups was 24%, 31% and 46% respectively. Individuals who received prednisolone for 20 weeks were significantly less likely to require additional steroid than those treated for 12. However this does not necessarily reflect clinical improvement. The decision to use additional prednisolone was left to the individual clinician's judgement at each of the six centres. It is not clear how consistency was ensured between individual physicians or at different stages of the trial. The protocol of the MP study was stringent in treating NFI. “Mild” deterioration in NFI and NFI of short duration were both treated. Any sustained (as little as one week) deterioration in monofilament testing at even a single test site was an indication for additional prednisolone and so a lower threshold for defining deterioration is likely to have been employed in the current study. This may in part account for the high proportion of individuals who received additional prednisolone. It is likely that some of the change labelled as deterioration was due to test response variability. In the TRIPOD 2 cohort 27% of prednisolone treated individuals with mild sensory impairment experienced deterioration necessitating additional prednisolone. A group with mild isolated sensory impairment would be expected to require less additional prednisolone than a group that included severe nerve impairment both sensory and motor and marked skin involvement.

The results of this small study should be interpreted with caution but it would appear that given the available data MP does not result in an increase in the number or severity of adverse events in individuals with leprosy in Nepal. However close detailed adverse event recording would still be required in any future studies of MP in this setting. The establishment of registries of corticosteroid treated patients at specialised centres could facilitate the collection of reliable adverse event data without the need to resort to more costly randomised controlled trials.

The clinical outcome of patients in the two arms of this study was not significantly different in terms of the validated clinical severity scale. The MP treated group had significantly less deterioration in sensory function during the 112 days of corticosteroid therapy but this was not maintained to the end of the 337 day follow-up period. This may be a reflection of the small numbers in the study, particularly towards the end of follow-up. A much larger study would be required to examine this potential effect further. However given the high proportion of individuals (who received MP) requiring additional prednisolone and the data published by Rao and colleagues[21] we do not think further clinical trials of high dose IV MP are warranted at present. Any future studies must also take into account the greater cost of giving intravenous treatment and its acceptability to patients.

This study has highlighted that corticosteroid treatment for T1R and NFI is sub-optimal even when given in large doses for 16 weeks. The majority of patients who experienced a “re-reaction” required additional prednisolone after the 16 week corticosteroid intervention had ended. It adds further support to the argument that treatment should be given for longer durations. Investigating risk factors for requiring additional prednisolone and the differences between those who have deterioration in symptoms whilst taking corticosteroids and those whose deterioration occurs later (or not at all) might enable clinicians to identify those individuals who might benefit from prolonged corticosteroid treatment at the outset. At present there is convincing evidence for corticosteroid regimes of at least 20 weeks [21] but some would argue for 24 weeks [22] and others even longer [23]. The development of more prolonged treatment protocols would require further monitoring of adverse events and in particular the long term sequelae of corticosteroid therapy. However studies with adequate power using improvement in nerve function as the primary outcome of the effect of corticosteroids and other agents need to be conducted.

## Supporting Information

Protocol S1 (0.16 MB DOC)

Checklist S1 Consort checklist
(0.22 MB DOC)

## References

[1] Moher D, Schulz KF, Altman DG (2001). The CONSORT statement: revised recommendations for improving the
quality of reports of parallel-group randomised trials.. Lancet.

[2] Lockwood DNJ, Burns DA, Breathnach SM, Cox NH, Griffiths CEM (2004). Leprosy.. Rook's Textbook of Dermatology 7th ed.

[3] WHO (2009). Global leprosy situation, 2009.. Wkly Epidemiol Rec.

[4] Ranque B, Nguyen VT, Vu HT, Nguyen TH, Nguyen NB (2007). Age is an important risk factor for onset and sequelae of
reversal reactions in Vietnamese patients with leprosy.. Clin Infect Dis.

[5] Marlowe SN, Hawksworth RA, Butlin CR, Nicholls PG, Lockwood DN (2004). Clinical outcomes in a randomized controlled study comparing
azathioprine and prednisolone versus prednisolone alone in the treatment of
severe leprosy type 1 reactions in Nepal.. Trans R Soc Trop Med Hyg.

[6] Richardus JH, Withington SG, Anderson AM, Croft RP, Nicholls PG (2003). Treatment with corticosteroids of long-standing nerve function
impairment in leprosy: a randomized controlled trial (TRIPOD
3).. Lepr Rev.

[7] van Brakel WH, Anderson AM, Withington SG, Croft RP, Nicholls PG (2003). The prognostic importance of detecting mild sensory impairment in
leprosy: a randomized controlled trial (TRIPOD 2).. Lepr Rev.

[8] Little D, Khanolkar-Young S, Coulthart A, Suneetha S, Lockwood DN (2001). Immunohistochemical analysis of cellular infiltrate and gamma
interferon, interleukin-12, and inducible nitric oxide synthase expression
in leprosy type 1 (reversal) reactions before and during prednisolone
treatment.. Infect Immun.

[9] Khanolkar-Young S, Rayment N, Brickell PM, Katz DR, Vinayakumar S (1995). Tumour necrosis factor-alpha (TNF-alpha) synthesis is associated
with the skin and peripheral nerve pathology of leprosy reversal
reactions.. Clin Exp Immunol.

[10] Weusten BL, Jacobs JW, Bijlsma JW (1993). Corticosteroid pulse therapy in active rheumatoid
arthritis.. Semin Arthritis Rheum.

[11] Filippini G, Brusaferri F, Sibley WA, Citterio A, Ciucci G (2000). Corticosteroids or ACTH for acute exacerbations in multiple
sclerosis.. Cochrane Database Syst Rev.

[12] Wandinger KP, Wessel K, Trillenberg P, Heindl N, Kirchner H (1998). Effect of high-dose methylprednisolone administration on immune
functions in multiple sclerosis patients.. Acta Neurol Scand.

[13] Youssef PP, Haynes DR, Triantafillou S, Parker A, Gamble JR (1997). Effects of pulse methylprednisolone on inflammatory mediators in
peripheral blood, synovial fluid, and synovial membrane in rheumatoid
arthritis.. Arthritis Rheum.

[14] Hirano T, Tsuboi N, Homma M, Oka K, Takekoshi T (2000). Comparative study of lymphocyte-suppressive potency between
prednisolone and methylprednisolone in rheumatoid arthritis.. Immunopharmacology.

[15] Ridley DS, Jopling WH (1966). Classification of Leprosy according to immunity.. Int J Lepr Other Mycobact Dis.

[16] Richardus JH, Withington SG, Anderson AM, Croft RP, Nicholls PG (2003). Adverse events of standardized regimens of corticosteroids for
prophylaxis and treatment of nerve function impairment in leprosy: results
from the ‘TRIPOD' trials.. Lepr Rev.

[17] Walker SL, Nicholls PG, Butlin CR, Nery JA, Roy HK (2008). Development and validation of a severity scale for leprosy type 1
reactions.. PLoS Negl Trop Dis.

[18] Brain (2000). Aids to the examination of the peripheral nervous
system..

[19] Pocaterra L, Jain S, Reddy R, Muzaffarullah S, Torres O (2006). Clinical course of erythema nodosum leprosum: an 11-year cohort
study in Hyderabad, India.. Am J Trop Med Hyg.

[20] Smith WC, Anderson AM, Withington SG, van Brakel WH, Croft RP (2004). Steroid prophylaxis for prevention of nerve function impairment
in leprosy: randomised placebo controlled trial (TRIPOD 1).. BMJ.

[21] Rao PS, Sugamaran DS, Richard J, Smith WC (2006). Multi-centre, double blind, randomized trial of three steroid
regimens in the treatment of type-1 reactions in leprosy.. Lepr Rev.

[22] Walker SL, Lockwood DN (2008). Leprosy type 1 (reversal) reactions and their
management.. Lepr Rev.

[23] Naafs B (2003). Treatment duration of reversal reaction: a reappraisal. Back to
the past.. Lepr Rev.

