# Supplementary material for: A Phase Two Randomised Controlled Double Blind Trial of High Dose Intravenous Methylprednisolone and Oral Prednisolone versus Intravenous Normal Saline and Oral Prednisolone in Individuals with Leprosy Type 1 Reactions and/or Nerve Function Impairment
Source: PLoS Negl Trop Dis. 2011 Apr 12;5(4):e1041. doi: 10.1371/journal.pntd.0001041 (PMC3075223; doi:10.1371/journal.pntd.0001041)
Supplement: Protocol S1 — (0.16 MB DOC) [file pntd.0001041.s001.doc]

**PROTOCOL**

**A Phase 2 trial to investigate the safety of early intravenous high dose methylprednisolone in acute neuritis and type 1 reactions (T1R) with neuritis.**

**Dr Rachel Hawksworth**

**Dr Diana Lockwood**

**Dr Murdo Macdonald**

**Dr Peter Nicholls**

**Dr Stephen Walker**

**Anandaban Hospital**

**PO Box 151**

**Kathmandu**

**Nepal**

**Clinical Research Unit, 2^nd^ Floor**

**Department of Infectious and Tropical Diseases**

**London School of Hygiene and Tropical Medicine**

**Keppel St**

**London**

**UK**

**WC1E 7HT**

**Email:** [**steve.walker@lshtm.ac.uk**](mailto:steve.walker@lshtm.ac.uk)

**drstevewalker@hotmail.com**

### Form 1- Eligibility

### Entry criteria

- Individuals with clinical evidence of T1R **with** new nerve function impairment (NFI). A T1R is clinically defined by the acute development of erythema and oedema of skin lesions, often accompanied by neuritis and oedema of the hands, feet and face. New NFI is defined as less than **6 months** duration of reduction in sensory, motor or autonomic function on history or examination.

**OR**

Individuals with new nerve function impairment without inflammation of skin lesions (**if** skin lesions are present)

- Aged 16-65
- Weigh more than 30Kg

### Exclusion criteria

- Anyone unwilling to give consent.
- **T1R without new NFI**.
- Individuals with severe active infection such as tuberculosis.
- Individuals with severe intercurrent disease (cardiac, hepatic or renal disorder)
- Contraindications to high dose methylprednisolone such as peptic ulcer disease, diabetes mellitus, glaucoma and uncontrolled hypertension or known allergy to methylprednisolone.
- Pregnant women and those females of child bearing capacity without at least one month of adequate contraception.
- Individuals who have taken systemic steroids or thalidomide within 3 months.
- Anyone unwilling to be admitted or return for follow-up.

### Form 2 – Informed consent

### Consent

- Trial carefully explained by investigator.
- Written explanatory note available in Nepali and English.
- Individual’s signature or mark obtained as proof of consent to take part in the trial.
- Individual’s signature or mark obtained as proof of consent to photography (participation in the trial is not dependent on giving consent for photography).
- Signature of enrolling researcher.
- **Attach adhesive label stating MP Study and the patient’s STUDY number to front of clinic notes.**
- **RECORD DATE, NAME ,CLINIC NUMBER, STUDY NUMBER AND CATEGORY (SEVERE OR MILD) IN THE METHYLPREDNISOLONE STUDY RECORD BOOK IN STUDY BOX**

### Form 3 - History and examination at registration

### Pre-treatment assessment

1. History
   - Date of onset of T1R
   - Symptoms of T1R (with particular attention to date of onset)
   - Leprosy classification and date of diagnosis
   - Leprosy treatment (type, starting and completion dates)
   - Time since completion of leprosy treatment
   - Previous history of T1R
2. Clinical Examination

- Full general clinical examination including T°, blood pressure and weight
- Leprosy clinical examination
  1. Nerves - signs and symptoms of neuritis

(pain, tenderness, enlargement)

- 1. Skin - location of lesions (body chart)

- type of lesions

(patches, plaques, papules, nodules)

- - - signs of inflammation in lesions
    - oedema of the hands and/or feet

**Form 4 -** **Sensory testing (ST)**

- Trigeminal*, ulnar, median and posterior tibial nerves on each side. The Purple 2g and Orange 10g Semmes-Weinstein monofilaments are used at 3 sites for each nerve on the hand (median and ulnar). The Orange 10g and Pink 300g monofilament at 3 sites for the posterior tibial nerves. (* cotton wool is used)
- Record on the diagram of the hands and feet the result of the monofilament testing at each test site using the following symbols

Purple 2g felt - ▲

Orange 10g felt - ■

Pink 300g felt - **#**

Neither monofilament felt – **A**

(Orange not felt on hands, Pink not felt on feet then mark an **A** at the site in question).

**Form 5** - **Voluntary motor testing (VMT)**

1. Facial, ulnar, radial, median and lateral popliteal nerves on each side. Using the modified MRC grading for muscle power.

**Facial nerve -** Forced eye closure (orbicularis oculi)

**Median nerve** - Thumb abduction (abductor pollicis brevis)

**Ulnar nerve** - Little finger abduction (abductor digiti minimi)

**Radial nerve** - Wrist extension (extensor muscles)

**Lateral popliteal nerve-** Foot dorsiflexion (tibialis anterior, peroneus longus and brevis)

### Testing procedure for each movement

The patient should be seated comfortably.

**Facial nerve -** Forced eye closure

- The patient is asked to close the eyes as tight as (s)he can.
- The tester tries to pull down the lower lid on both sides using his/her thumbs

**Median nerve** -Thumb abduction

- The wrist is held in extension and the patient is asked to lift his thumb up.
- Pressure is applied over the lateral side of the base of the proximal phalanx.

**Ulnar nerve** - Little finger abduction

- Ask the patient to abduct the little finger with MCP in slight flexion.
- Pressure is applied over the base of the proximal phalanx.

**Radial nerve** - Wrist extention

- Ask the patient to make a fist and lift the wrist up.
- Pressure is applied over the dorsum of hand.

**Lateral popliteal nerve -** Foot dorsiflexion

- Ask the patient to lift the foot up.
- Pressure is applied over the dorsum of foot.

1. Score is derived for each nerve. MRC = 5 scores 0

MRC = 4 scores 1

MRC = 3 scores 2

MRC < 3 scores 3

If there is evidence of NFI for a given nerve then confirmation of the duration of the NFI should be sought from the affected individual to determine whether or not this is new.

| **MRC modified grading of muscle power** | | **Severity Scale Score** |
| --- | --- | --- |
| Score | Muscle response |
| 5 | Full range of movement (FROM) | 0 |
| 4 | FROM but less than normal resistance | 1 |
| 3 | FROM but no resistance | 2 |
| 2 | Partial range of movement with no resistance | 3 |
| 1 | Perceptible contraction of the muscle not resulting in joint movement | 3 |
| 0 | Complete paralysis | 3 |

## Form 7

## Pre-enrolment Screening Tests

- Full clinical examination.
- Full blood count, creatinine, random blood sugar.
- Stool specimen will be examined for ova, cysts and parasites
- Chest radiograph
- Sputum examination for AFB
- Pregnancy test

## Staging and initial trial investigations

- Skin smears from four sites including both ear lobes and two active skin lesions (the elbow or thigh should be used if there is only one skin lesion and both should be used if there are none).Smears are unnecessary if they have been done within 3 months of enrolment into the trial.
- 6mm punch biopsy of skin for Ridley Jopling classification if not already done.
- 6mm punch biopsy of skin at baseline. The site of biopsy should be clearly documented on **FORM 8** to enable subsequent biopsies to be taken from an adjacent site. Ulcerated lesions should be avoided if possible. **USE PLAIN 1 0R 2% LIGNOCAINE DO NOT USE LIGNOCAINE WITH ADRENALINE**. The skin biopsy should be halved **lengthwise**. Place half in the test vials with RNA*later*, the other half should be placed in a dry test vial. A yellow top should be placed in the cap of the vial containing the RNA*later*. These should be taken by the **doctor** to the laboratory and handed to the member of laboratory staff responsible
- 5 ml heparinised venous blood specimen for trial at baseline.

## Subsequent trial investigations

- 5ml heparinised venous blood specimens at days 3, 14, 28 and weeks 16 and 24.
- 6mm punch biopsy of skin at day 4 and week 16 taken from same site as baseline specimen.

**Admission**

- All those enrolled in the trial will be admitted for the infusions (either methylprednisolone or placebo)
- Period of admission is 7-14 days for uncomplicated cases

**Allocation of treatment**

**Preparatory actions.**

A Randomisation Table has been prepared specifying the order in which individuals recruited to the study are to be allocated to MP and P arms. This lists allocations within two separate lists, one for individuals with severe reactions and one for individuals with mild reactions, as clinically assessed at intake. Each list has been generated using random numbers in an Excel spreadsheet and includes a sequence number. In each, the randomisation is organised so that, among each block of four individuals recruited to the study, there are two allocations to the MP arm and two the P arm. Example(not the actual allocations):

| **Seq No.** | **Severe Reaction List** | **Seq No.** | **Mild Reaction List** |
| --- | --- | --- | --- |
| 1 | MP | 1 | MP |
| 2 | MP | 2 | Placebo |
| 3 | Placebo | 3 | Placebo |
| 4 | Placebo | 4 | MP |
|  |  |  |  |
| 5 | Placebo | 5 | MP |
|  | Etc. |  | Etc. |

If this were the true allocation, the first recruited individual with a severe reaction would be allocated to the MP arm, the second also to the MP arm, the third to the Placebo arm etc.

The randomisation allows for up to 40 allocations of individuals with severe reactions and up to 40 individuals with mild reactions. The total recruitment to the study will be 60, 30 in each arm.

At this stage, the relative frequency of mild and severe reactions is unknown and no target has been set for the numbers of mild or severe reactions.

**Allocation process**

To ensure blinding of clinicians to allocation to treatment arms, the following procedure is to be followed.

Two designated individuals in the hospital pharmacy will need to provide prednisolone or placebo tablets and normal saline or methylprednisolone infusions during the first three days after enrolment to the trial. We propose this be the responsibility of just one senior pharmacist and that this individual alone have access to information about the allocation to treatment arms. It should be the responsibility of this individual to maintain a record of recruitment and to deliver appropriate tablets and infusions to individual patients in the hospital ward. This will be achieved as follows:

From the lists in the Randomisation Table we have prepared duplicate sets of 80 labelled and sealed envelopes containing the details of how each individual recruited to the study is to be allocated to Placebo and MP arms. Each envelope and its contents will be linked to the Randomisation Table by the details of reaction severity and sequence number.

The labelling on each envelope will identify the reaction severity group – Mild or Severe – and the sequence number within the group – 1 to 40. The labelling will NOT specify the treatment allocation (See Appendix One).

Inside the sealed envelopes will be placed a one page form with three sections with contents as follows (See Appendix Two):

Part One describes the allocated treatment arm, either methyl prednisolone or placebo.

Part Two provides space for the pharmacist to record the patients details.

Part Three guides the pharmacist as to the tablets and infusion to be collected from pharmacy and delivered to the patient in the ward on each of the first three days from intake.

The full set of sealed envelopes will be handed to the designated pharmacist at the start of the study. They are to be kept in a secure place. No other staff should have access to the envelopes. Only the pharmacist is permitted to open the envelopes.

When a clinician identifies an individual to be enrolled in the study he will request the pharmacist to register this individual and identify the reaction group as mild or severe. The pharmacist will then take the following actions:

Register the new case on a master list held in the pharmacy, recording details of hospital card number, name and registration date. This list will have the format following:

| **Severe Reaction Group** | | | | **Mild Reaction Group** | | | |
| --- | --- | --- | --- | --- | --- | --- | --- |
| **Envelope Number** | **Hospital Number** | **Patient Name** | **Date** | **Envelope Number** | **Hospital Number** | **Patient Name** | **Date** |
| **1** |  |  |  | **1** |  |  |  |
| **2** |  |  |  | **2** |  |  |  |
| **3** |  |  |  | **3** |  |  |  |
| **4** |  |  |  | **4** |  |  |  |
| **5** |  |  |  | **5** |  |  |  |
| **Etc** |  |  |  | **Etc** |  |  |  |

The pharmacist will then give the sequence number/envelope number to the clinician so that this information can be recorded on the appropriate data form – FORM 3 and ultimately be entered onto computer (e.g. Severe Reaction Group #1).

This is an essential action that will ensure that the treatment arm can be identified on the computerized database.

The pharmacist will then retrieve the envelope containing the form specifying the treatment arm for the designated sequence number and reaction severity group. He/she will open the envelope, note the allocation to a treatment arm and file the form in a temporary location designated for individuals under process (i.e. covering the first three days from admission when MP/saline is being given).

He/she will then proceed to pharmacy, collect the appropriate infusion and tablets and deliver them to the patient in the hospital ward. The labelling on both tablets and infusion should NOT specify contents other than “Treatments relating to MP study”.

The pharmacist will repeat these actions on the second and third days.

After the third day he/she will record the delivery of tablets and infusion as completed and transfer the form to a permanent file held in a secure place.

Access to files or forms by any of the clinical staff during the course of the study is denied.

**Other points**

The pharmacists must be absolutely certain to assign the correct envelope number and to provide the specified tablets and infusion on each of the first days following registration. This is fundamental to the success of the research.

A second person from the pharmacy should be involved to try and ensure that no mistakes are made.

In the event of some clinical emergency during the first 96 hours after registration of each new patient it may be necessary to ascertain if the patient concerned received MP. To provide access to this information, a second set of sealed envelopes, identical to the first, will be provided. These will be in the safe-keeping of the project director. Should the need arise, he may open the envelope relating to the individual concerned.

## Treatment

- All individuals will receive **albendazole 400mg** daily for three days at enrolment.
- All individuals will receive **famotidine** **40mg** daily for whilst on steroids.
- If the stool sample demonstrates *Entamoeba histolytica* then Metronidazole/Diloxanide furoate (**Metrin DF**) should be prescribed for 5 days
- Steroid protocol:

**Methylprednisolone/prednisolone arm ≡ 6.15g of prednisolone**

**Assessment 1** Day 1 IV methylprednisolone 1g (in 100ml Normal Saline)

+ **placebo** **tablets**

Day 2 IV methylprednisolone 1g (in 100ml Normal Saline) + **placebo tablets**

Day 3 IV methylprednisolone 1g (in 100ml Normal Saline) + **placebo tablets**

**Assessment 2** Day 4-7 prednisolone 40mg

**Assessment 3** Week 2 prednisolone 40mg

**Assessment 4** Week 3 prednisolone 35mg

Week 4 prednisolone 35mg

**Assessment 5** Week 5 prednisolone 30mg

Week 6 prednisolone 30mg

Week 7 prednisolone 25mg

Week 8 prednisolone 25mg

**Assessment 6** Week 9 prednisolone 20mg

Week 10 prednisolone 20mg

Week 11 prednisolone 15mg

Week 12 prednisolone 15mg

**Assessment 7** Week 13 prednisolone 10mg

Week 14 prednisolone 10mg

Week 15 prednisolone 5mg

Week 16 prednisolone 5mg

**Assessment 8** Week 17 Off steroids

### Prednisolone alone arm = 2.52g of prednisolone

**Assessment 1** Day 1 IV placebo (100ml Normal Saline) + prednisolone 40mg

Day 2 IV placebo (100ml Normal Saline)+ prednisolone 40mg

Day 3 IV placebo (100ml Normal Saline)+ prednisolone 40mg

**Assessment 2 D**ay 4-7 prednisolone 40mg

**Assessment 3** Week 2 prednisolone 40mg

**Assessment 4** Week 3 prednisolone 35mg

Week 4 prednisolone 35mg

**Assessment 5** Week 5 prednisolone 30mg

Week 6 prednisolone 30mg

Week 7 prednisolone 25mg

Week 8 prednisolone 25mg

**Assessment 6** Week 9 prednisolone 20mg

Week 10 prednisolone 20mg

Week 11 prednisolone 15mg

Week 12 prednisolone 15mg

**Assessment 7** Week 13 prednisolone 10mg

Week 14 prednisolone 10mg

Week 15 prednisolone 5mg

Week 16 prednisolone 5mg

**Assessment 8** Week 17 Off steroids

**Form 6 - Assessment during study and after finishing prednisolone**

- Full general clinical assessment (including temperature, blood pressure and weight) at days 4, 8, 15, 29 and 4 weekly thereafter for 48 weeks.
- Leprosy clinical examination at days 4, 8, 15, 29 and 4 weekly thereafter for 48 weeks.
- Nerve function tests (ST and VMT) at days 4, 8, 15, 29 and 4 weekly thereafter for 48 weeks.
- Severity score at days 4, 8, 15, 29 and 4 weekly thereafter for 48 weeks.
- Any clinical examinations that are not part of the trial schedule should be recorded on a separate form – **Form 8**. Additional forms are kept in the protocol file in the study box.
- The date, reason and outcome of any additional examinations should also be recorded.
- **RECORD THE DATE AND NUMBER OF PATIENT’S NEXT ASSESSMENT IN THE DIARY IN THE STUDY BOX**

### Safety monitoring

- Specific questioning at each visit with respect to adverse events or new symptoms possibly related to trial interventions.
- Major adverse events

1. Gastrointestinal bleeding
2. Nocturia, polyuria, polydipsia
3. Diabetes mellitus
4. Psychosis or other mental health problems
5. Weight loss >5kg
6. Weight gain
7. Glaucoma
8. Cataract
9. Hypertension >160/90 on two separate readings at least one week apart
10. Infections
11. Infected ulcers
12. Corneal ulcer
13. Tuberculosis
14. Night sweats

- Minor adverse events
  1. Moon face
  2. Acne
  3. Cutaneous (including nails)fungal infections
  4. Gastric pain requiring antacids

## Criteria for unblinding

- In the event of a major adverse event in the first 96 hours which is felt could be related to methylprednisolone then the code can be broken for that individual in order to aid management of the problem.

**Recurrence of T1R OR neuritis**

- Criteria for using additional prednisolone

1. Sustained deterioration for a period of at least two weeks of:
   1. Deterioration in nerve function
   2. Nerve pain unresponsive to analgesics
   3. Palpable swelling of skin patches
   4. New erythematous and raised skin patches
2. Deterioration in nerve function which the study doctors believe requires immediate additional prednisolone

- The patient must be examined by at least two of the study doctors and they should be in agreement about giving the patient additional prednisolone.
- The reasons for the additional prednisolone and the date started should be recorded.

## Regimen for additional prednisolone

- If there is recurrence of T1R with NFI (or nerve pain unresponsive to analgesics) on treatment then add extra prednisolone to make up a total of 40mg and then taper according to the original regimen.
- If there is recurrence of T1R with skin signs but **no** NFI then:
  1. If recurrence within the first ten weeks of treatment or there is facial involvement then add extra prednisolone to make up a total of 40mg and then taper according to the original regimen.
  2. If recurrence after ten weeks of treatment then add extra prednisolone to make up a total of 20mg and then taper according to the original regimen.

**Monitoring the trial**

- Drs KV Krishna Moorthy and PS S Sundar Rao who are independent of the conception, design and management of the trial have agreed to act as trial monitors.

## Data entry

- Each subject enrolled into the study will have an individual case booklet for recording of all clinical and laboratory data.
- An anonymised Access database will be created for storage of trial data which will subsequently be analysed using standard statistical packages.

**Late Clinic Attendances**

If a trial subject does not attend a scheduled assessment then they will be contacted and asked to come to the next clinic for their assessment. It is essential that the date of the attendance is recorded. **The number of the Assessment should not be changed regardless of how late the assessment is carried out.**

The next assessment after this should be scheduled as though the original assessment had been performed as planned. If the assessment is so late that the following assessment has also been missed then the next assessment should be scheduled for 28 days (four weeks) later.

**If a participant has missed certain trial investigations then these should be performed when they next attend.**

**Unscheduled Clinic Attendances/examinations**

- All unscheduled examinations (if an inpatient) or clinic attendances should be recorded on Form 8.
- If the reason for the attendance/examination is related to their leprosy diagnosis then Forms 4,5 and 6 should also be completed.
- All Forms should be completed in red ink or clearly marked at the top UNSCHEDULED.
- The **next** assessment number should be used for unscheduled visits/examinations.
- It should be documented if the clinician feels the attendance is related to corticosteroids.

**Non reactional controls**

Borderline patients who are undergoing a diagnostic skin biopsy should be approached to see if they would consent to a 6mm skin punch biopsy done at the same time as their diagnostic one.

They should be guided through the specific information leaflet and if agreeable asked to sign the consent form for the additional biopsy. The aim is to recruit 10 controls.

**THESE CONTROLS MUST NOT BE IN REACTION.**

- **CHECKLIST ON ADMISSION**

**Type 1 reaction with nerve function impairment or neuritis less than 6 months duration.**

**Reaction mild or severe**

**Inform pharmacy of admission and reaction type**

**Physical examination**

**FBC, creatinine, random glucose**

**Stool sample**

**Chest Xray**

**Sputum**

**Pregnancy test females with childbearing capacity**

**Appropriate contraception in females with childbearing capacity**

**Prescribe albendazole and famotidine**

**Intravenous access**

**REQUIREMENTS AT EACH VISIT**

**Assessment 1 (Enrolment/Admission)**

**Form 1 Eligibility**

**Form 2 Consent**

**Form 3 History and examination**

**Form 4 Sensory testing**

**Form 5 Motor testing**

**Form 7 Investigations**

**FBC, Creatinine, random blood glucose**

**5ml of venous blood collected in heparin (for whole blood assay and Luminex)**

**Stool specimen**

**Chest Xray**

**Sputum**

**Pregnancy test**

**Contraception**

**Albendazole and famotidine**

**Skin smears if not done in previous 3 months**

**Skin biopsy for Ridley Jopling classification if not done at a previous attendance**

**Skin biopsy from the edge of an area of reactional (non-ulcerated) skin**

**Inform pharmacy of clinical categorisation of the reaction – severe or not severe**

**Intravenous access**

**Infusion duration 1 hour**

**Take oral prednisolone or placebo at same time**

**Assessment 2 (Day 4- the day after the last infusion)**

**Form 4**

**Form 5**

**Form 6**

**Form 7**

**5ml of venous blood collected in heparin (for whole blood assay and Luminex)**

**Skin biopsy from the edge of an area of reactional (non-ulcerated) skin**

**Assessment 3 (Day 8 after one week of steroids)**

**Form 4**

**Form 5**

**Form 6**

**Assessment 4 (Day 15 after two weeks of steroids)**

**Form 4**

**Form 5**

**Form 6**

**Form 7**

**5ml of venous blood collected in heparin (for whole blood assay and Luminex)**

****REMOVE BIOPSY SUTURES****

**Assessment 5 (Day 29 after four weeks of steroids)**

**Form 4**

**Form 5**

**Form 6**

**Form 7**

**5ml of venous blood collected in heparin (for whole blood assay and Luminex)**

**Assessment 6 (Day 57 after eight weeks steroids)**

**Form 4**

**Form 5**

**Form 6**

**Assessment 7 (Day 85 after 12 weeks steroids)**

**Form 4**

**Form 5**

**Form 6**

**Assessment 8 (Day 113 after 16 weeks steroids)**

**Form 4**

**Form 5**

**Form 6**

**Form 7**

**5ml of venous blood collected in heparin (for whole blood assay and Luminex)**

**Skin biopsy from an area of reactional (non-ulcerated) or previously reactional skin**

**STOP STEROIDS IF NO DETERIORATION**

**Assessment 9 (Day 141 after 20 weeks in trial)**

**Form 4**

**Form 5**

**Form 6**

**Assessment 10 (Day 169 after 24 weeks in trial)**

**Form 4**

**Form 5**

**Form 6**

**Form 7**

**5ml of venous blood collected in heparin (for whole blood assay and Luminex)**

**Assessment 11 (Day 197 after 28 weeks in trial)**

**Form 4**

**Form 5**

**Form 6**

**Assessment 12 (Day 225 after 32 weeks in trial)**

**Form 4**

**Form 5**

**Form 6**

**Assessment 13 (Day 253 after 36 weeks in trial)**

**Form 4**

**Form 5**

**Form 6**

**Assessment 14 (Day 281 after 40 weeks in trial)**

**Form 4**

**Form 5**

**Form 6**

**Assessment 15 (Day 309 after 44 weeks in trial)**

**Form 4**

**Form 5**

**Form 6**

**Assessment 16 (Day 337 after 48 weeks in trial)**

**Form 4**

**Form 5**

**Form 6**

**USE OF THE TRIAL FORMS**

**Form 1**

Used to screen and register patients at the first visit

**Form 2**

Consent form for the MP study.

**Form 3**

Initial history and examination for admission

**Form 4**

Monofilament sensory testing

**Form 5**

Voluntary motor testing

**Form 6**

Follow up assessments including documentation of the current prednisolone dose and any additional prednisolone that may be required.

**Form 7**

Recording any investigations performed at an assessment.

**Form 8**

Information that might need to be recorded but is not covered by the other trial documentation. It should also be used to document skin biopsy procedure and site.

It should also be used if a participant withdraws and **wishes** to give a reason.

**TRIAL SPECIMENS**

**Skin biopsies**

- 6mm punch biopsies taken from the edge of an active non–ulcerated skin lesion should be bisected.
- Half is to be placed in a cryogenic vial with a WHITE cap and snap frozen in liquid nitrogen.
- The other half is to be placed together with RNA*later* in a cryogenic vial with a YELLOW cap. This should be stored overnight at 4°C to allow penetration of the RNA*later*. The excess should be removed the following day and the specimen in the vial stored at -80°C.
- Each vial should be clearly labelled with the participants unique study code and the date.

**Venous blood samples**

- These should be collected into heparin. 5ml is required.
- The stimulation is undertaken and the supernatant removed and frozen at -70°C after 24 hours with the exception of the DAY 4 (if taken on a Saturday) sample which will be stimulated for 48 hours.

**Appendix One**

**Envelope labelling**

| **Methyl Prednisolone Study**  Reaction type:  Sequence Number: |
| --- |

**Appendix Two**

**Form enclosed in Envelope**

**Version One – for Methylprednisolone arm**

|  | **Severity Group**: |  |
| --- | --- | --- |
|  | **Sequence Number** |  |
| **Part One** | **Study Arm** | **Methylprednisolone** |
| **Part Two** | **Patient details**:  Name  Clinic Card  Study Number  Enrolment date: |  |
| **Part Three** |  |  |
| **Day 1** | **8 placebo tablets**  **1 g Methylprednisolone IV** | **Completed by**: |
| **Day 2** | **8 placebo tablets**  **1g Methylprednisolone IV** | **Completed by**: |
| **Day 3** | **8 placebo tablet**  **1 g Methylprednisolone IV** | **Completed by**: |

**Version Two – for Placebo arm**

|  | **Severity Group**: |  |
| --- | --- | --- |
|  | **Sequence Number** |  |
| **Part One** | **Study Arm** | **Placebo** |
| **Part Two** | **Patient details**:  Name  Clinic Card  Study Number  Enrolment date: |  |
| **Part Three** |  |  |
| **Day 1** | **40mg prednisolone tablets**  **IV Normal Saline** | **Completed by**: |
| **Day 2** | **40mg prednisolone tablets**  **IV Normal Saline** | **Completed by**: |
| **Day 3** | **40mg prednisolone tablets**  **IV Normal Saline** | **Completed by**: |
